# Association between leaflet fusion pattern and thoracic aorta morphology in patients with bicuspid aortic valve

**DOI:** 10.1186/1532-429X-14-S1-M4

**Published:** 2012-02-01

**Authors:** Bryce Merritt, Alexander Turin, Michael Markl, James Carr

**Affiliations:** 1 Radiology, Northwestern University, Feinberg School of Medicine, Chicago, IL, USA; 2 Biomedical Engineering, Northwestern University, Chicago, IL, USA; 3 Bluhm Cardiovascular Institute, Northwestern Memorial Hospital, Chicago, IL, USA

## Background

Bicuspid Aortic Valve (BAV) is the most common congenital heart defect and is associated with a high risk of aortic dilation. We sought to determine if patients with certain BAV phenotypes are predisposed to particular morphological abnormalities of the thoracic aorta.

## Methods

One hundred and ninety-two patients with BAV who underwent magnetic resonance angiography between January 2007 and July 2010 were retrospectively identified. Aortic morphology was examined through measurements of diameter at nine levels along the thoracic aorta, 3D volume of the ascending aorta, vessel eccentricity, and assessment of aortic root and arch morphology. Diameters and volumes were normalized to body surface area. BAV phenotypes were defined by fusion of the right and left coronary cusps (Type 1), right and non-coronary cusps (Type 2), and left and non-coronary cusps (Type 3).

## Results

We found 140 patients (73%) with Type 1 fusion, 46 patients (24%) with Type 2 fusion, and 6 patients (3%) with Type 3 fusion. Mean diameter at the sinus of Valsalva was significantly larger in Type 1 patients (1.97 vs. 1.77 cm/m2; p<0.001). Mean aortic volume in the proximal ascending aorta was significantly greater in Type 2 patients (0.93 vs. 0.60 cm3/m2; p<0.01). Type 2 patients possessed greater dimensions in the distal ascending aorta and proximal aortic arch, and were also more likely to have ascending distention of the aortic root.

## Conclusions

Our results suggest that BAV with Type 1 fusion is associated with dilation of the aortic root, while BAV with Type 2 fusion is associated with dilation of the distal ascending aorta and proximal arch. Our findings support the role of hemodynamics in the aortic dilation seen in the presence of BAV, and illustrate the morphological heterogeneity that exists among BAV phenotypes.

## Funding

Medical Student Summer Research Grant through Northwestern University's Feinberg School of Medicine.

**Figure 1:**
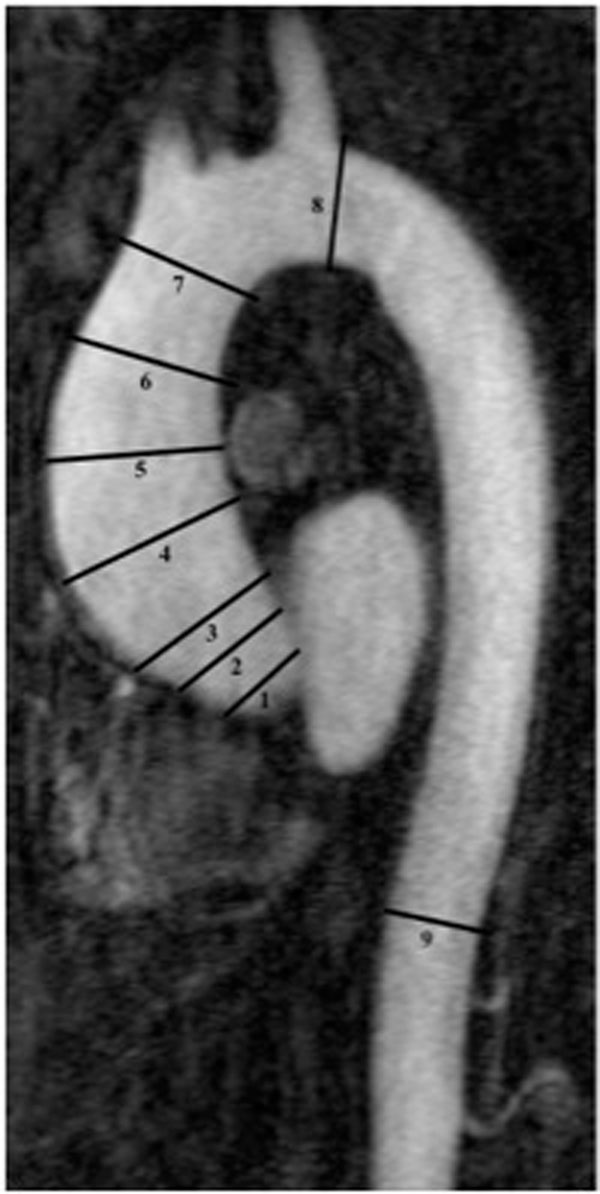
Cardiac magnetic resonance image annotated to illustrate the 9 levels at which the aortic diameter was measured. 1. Aortic annulus; 2. Sinus of Valsalva; 3. Sinotubular junction; 4. Proximal ascending aorta; 5. Mid-ascending aorta; 6. Distal ascending aorta; 7. Proximal to the innominate trunk (proximal aortic arch); 8. Distal to the left subclavian artery (distal aortic arch); 9. Descending aorta at the level of the diaphragm.

**Figure 2:**
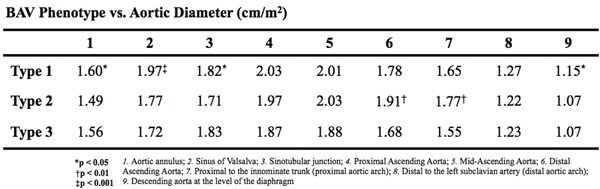
Variations in aortic diameter with bicuspid aortic valve fusion pattern type.

